# Metabolic Syndrome in Patients with Chronic Obstructive Pulmonary Disease in Medicine Department of a Tertiary Care Hospital: A Descriptive Cross-sectional Study

**DOI:** 10.31729/jnma.6410

**Published:** 2021-04-30

**Authors:** Niraj Kumar Singh, Lochan Karki

**Affiliations:** 1 Department of Internal Medicine, Nepal Medical College-Teaching Hospital, Jorpati, Kathmandu, Nepal; 2 Department of Medicine, National Academy of Medical Sciences, Kathmandu, Nepal

**Keywords:** *cachexia*, *co-morbidities*, *COPD*, *metabolic syndrome*, *systemic inflammation*

## Abstract

**Introduction:**

The best recognized systemic manifestations of chronic obstructive pulmonary disease include, cardiovascular co-morbidities, cachexia and muscle dysfunction, osteoporosis, anemia, and clinical depression and anxiety. This study was undertaken to find the prevalence of the metabolic syndrome in chronic obstructive pulmonary disease patients who were admitted in the medicine department of a tertiary care hospital.

**Methods:**

A descriptive cross-sectional study was carried out in the medicine department of Nepal Medical College and teaching hospital between October 2009 and January 2010. Ethical approval was taken from the Intitutional Review Committee. Convenience sampling technique was used. All chronic obstructive pulmonary disease patients were included. Descriptive statistics were used to evaluate baseline characteristics. Point estimate at 90% Confidence Interval was calculated along with frequency and proportion for binary data.

**Results:**

Out of 84 patients, the period prevalence of metabolic syndrome is 30 (35.71%) (29.80-40.20 at 90% Confidence Interval) as per the definition by International Diabetes Federation 2006 for South Asians. Among them, 35 (41.67%) were male and 49 (58.33% ) were female. Eight (9.5%) were of age between 40-49 years, 11 (13.1%) between 50-59 years, 27 (32.1%) between 60-69 years and 38 (45.2%) of 70 years and above.

**Conclusions:**

The study showed that the prevalence of metabolic syndrome was found to be lower than the previous study done in similar settings.

## INTRODUCTION

Chronic obstructive pulmonary disease (COPD) is one of the most prevalent diseases, expected to move to the 3rd leading cause of mortality in 2020.^1^ It is characterized by poorly reversible and progressive airflow limitation and is associated with an abnormal inflammatory response of the lungs to noxious particles or gases, particularly cigarette smoke.^2^

The best recognized systemic manifestations of COPD include cardiovascular co-morbidities, cachexia and muscle dysfunction, osteoporosis, anemia, and clinical depression and anxiety.^3,4^ A study in 2004 reported increased incidence of metabolic syndrome among COPD patients.^5^ They reported that 47% of COPD patients presented 3 or more determinants of the metabolic syndrome.^5^ In 2009 another study also reported increased incidence of metabolic syndrome among COPD patients.^6^ They have reported that the frequency of the metabolic syndrome in patients with chronic bronchitis, Global Initiative for Chronic Obstructive Lung Disease (GOLD) stages I, II, III, and IV were 53%, 50%, 53%, 37%, 44%, respectively.^6^

This study was undertaken to find the prevalence of the metabolic syndrome in COPD patients who were admitted in the department of medicine of Nepal Medical College and Teaching Hospital.

## METHODS

A descriptive cross-sectional study was carried out in the medicine department of Nepal Medical College and teaching hospital (NMCTH) between October 2009and January 2010. The study was approved by the institutional review committee (IRC) of Nepal Medical College and Teaching hospital, and participants gave their informed consent. The sample size calculation was done as below:

$$\begin{array}{ll} \text{n} & ={\text{Z}}^{\text{2}}\times \text{p}\times \text{q}/{\text{e}}^{\text{2}}\\ & ={\left(1.645\right)}^{2}\times 0.5\times (1-0.5)/{\left(0.1\right)}^{\text{2}}\\ & =67 \end{array}$$

where,

Z = 1.645 at 90% Confidence Interval (CI)
p = prevalence of metabolic syndrome in COPD patients in previous study, 47%^5^
q = (1-p)
e = margin of error, 10%

Therefore, the calculated sample size was 67. Adding the 10% non-response rate, the sample size that should have been taken was 74. However, 84 samples were taken. Convenience sampling technique was used.

All COPD patients in stable clinical condition, with no exacerbation of their disease and/or use of systemic corticosteroid in the preceding 3 months with age more than 40 years were included in the study. The diagnosis of COPD was based on current or past smoking history, history of productive cough for at least 3 months for consecutive 2 years, exposure to biofuel, clinical evaluation, and pulmonary function tests. They were classified according to the GOLD. The metabolic sydrome was diagnosed as per the new international diabetes federation (IDF) definition 2006 which states that for a person to be defined as having the metabolic syndrome; they must have: central obesity plus any two of following four factors: raised triglycerides, raised blood pressure, reduced HDL cholesterol and raised fasting plasma glucose.^7^

Blood pressure (BP) measurements were taken according JNC 7 recommendations.^8^ The auscultatory method of BP measurement was used. Serum glucose concentration and lipid profile were measured by an enzymatic colorimetric assay in laboratory of Nepal Medical College and teaching hospital. Pulmonary function test were obtained of all COPD Patients to determine FVC, FEV1, FVC/FEV1 ratio and percentage of predicted FEV1 in Nepal Medical College and teaching hospital. Pulmonary function test (PFT) was performed by Spiro232, Morgan.

Data analysis was performed with the Statistical Package for Social Science (SPSS; Chicago, IL), version 18.0. Descriptive statistics were used to measure baseline characteristics. Point estimate at 90% CI was calculated along with frequency and proportion for binary data.

## RESULTS

Out of 84 patients with COPD, the period prevalence of metabolic syndrome is 30 (35.71%) (29.80-40.20 at 90% Confidence Interval) as per the definition by IDF 2006 for South Asians.

Among 84 patients, 35 (41.67%) were male and 49 (58.33%) were female. Eight (9.5%) were of age between 40-49 years, 11 (13.1%) between 50-59 years, 27 (32.1%) between 60-69 years and 38 (45.2%) of 70 years and above. The mean age of patients in COPD group was 67.13±10.76 years with mean abdominal circumference of 77.46±10.43 cm, mean TG of 154.37 ±44.96 mg/dl, mean HDL of 44.3±8.47 mg/dl. Twenty people were hypertensive with mean systolic BP of 125.83±19.91 mm of Hg with mean diastolic BP of 76.48±11.43 mm of Hg. Thirty patients in COPD group had excess abdominal circumference, whereas 33 patients in COPD group had excess triglyceride level (Table 1).

**Table 1:** Distribution of different components of metabolic syndrome in COPD patients.

Features	Frequency n (%)
Excess abdominal circumference (>90 cm in males and >80 cm in females)	
Male	14 (40.00)
Female	16 (32.65)
Total	30 (35.71)
TG>150 mg/dl	
Male	17 (48.57)
Female	16 (32.65)
Total	33 (39.28)
Low HDL (<40 mg/dl in males & <50 mg/dl in females)	
Male	15 (42.86)
Female	13 (26.53)
Total	28 (33.33)
DM	
Male	3 (8.58)
Female	7 (14.28)
Total	10 (11.90)
Hypertension	
Male	10 (28.57)
Female	10 (20.40)
Total	20 (23.80)

**Figure 1.**
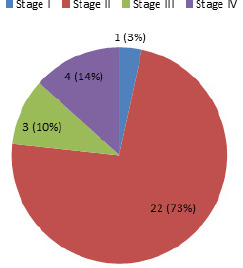
Distribution of metabolic syndrome cases according to GOLD stage I, II, III and IV.

Among 30 cases with metabolic syndrome in COPD group, 14 (40.0%) were male and 16 (32.65%) were female, whereas the distribution of metabolic syndrome cases according to GOLD stage I, II, III and IV were as given below (Figure 1).

Mean HDL level in Stage I was 41.0mg/dl and the corresponding values for Stage II, III, and IV were 44.07 mg/dl, 44.36 mg/dl and 45.85 mg/dl respectively. Mean TG level in Stage I was 217.0 mg/dl and the corresponding values for Stage II, III, and IV were 156.07 mg/dl, 147.09 mg/dl and 143.31 mg/dl respectively. Mean FBS in Stage I, II, III and IV were 108.5 mg/dl, 96.12 mg/dl, 88.45 mg/dl and 81.00 mg/dl respectively. Mean SBP in Stage I, II, III and IV were 120 mmHg, 128 mmHg, 118 mmHg and 121 mmHg respectively.

## DISCUSSION

Evaluating for risk factors, it was found in the present study that every cases of COPD had a strong history of smoking. Biofuel was also found to be a second risk factor in about 51% of cases, among them maximum were female residing in rural areas of Nepal.

Karine Marquis, et al. has reported increased incidence of metabolic syndrome among COPD patients.^9^ They have reported that 47% of COPD patients presented 3 or more determinants of the metabolic syndrome as compared to 21% of control participants. They did their studies in Caucasians population. But, the present study included a mixture of Nepalese Aryans and Mongolians. Indeed, the metabolic syndrome varies substantially among ethnic groups and this difference has been reflected by the difference of prevalence in this study as compared to theirs.

Predisposition to metabolic syndrome and systemic inflammation in COPD can be considered owing to various explanations. COPD is characterized by chronic hypoxia. This results in systemic hypoxia as well as local inflammatory response within adipose tissue per se, and may contribute to elevations in circulatory mediators by spillover from the adipose tissue to the systemic compartment leading to systemic inflammation. Although the extent to which adipose tissue production and release of inflammatory cytokines contributes to the chronic systemic inflammatory syndrome in COPD is not yet well defined, several stimulating ideas can be derived from experimental studies aimed to unravel the effects of hypoxia in adipocyte cell cultures, from animal hypoxic models, and from disorders other than COPD.

Physical inactivity and existing metabolic syndrome are also independently related to systemic inflammation in patients with COPD.^10^

The present study showed the prevalence of metabolic syndrome goes on decreasing with the increase in severity of COPD as indicated by GOLD stage. The frequency of the metabolic syndrome in patients with GOLD stage I, II, III, and IV was found to be 50%, 37.93%, 30.00% and 28.57% respectively (total, 35.71%).

It has also been found that there is an increasing trend for HDL level with increase in severity of COPD as per GOLD stage. Mean HDL level in GOLD stage IV was 45.85 mg/dl as compared to 41.00 mg/dl in GOLD stage I.

Tisi, et al. reported a similar finding and hypothesized that the increased work of breathing might constitute a chronic exercise stimulus for the respiratory muscles, resulting in an increase in HDL levels. Although possible, it is doubtful that respiratory muscles may have such a systemic impact.^11^

In 2009, 5th October, a joint interim statement for diagnosis of metabolic syndrome was given by ‘International Diabetes Federation Task Force on Epidemiology and Prevention; National Heart, Lung, and Blood Institute; American Heart Association; World Heart Federation; International Atherosclerosis Society; and International Association for the Study of Obesity’. As a common consensus, they formulated criteria for metabolic syndrome in which increased abdominal circumference was no more a prerequisite but one of the components of metabolic syndrome.

This change made no significant difference in diagnosis of metabolic syndrome as compared to those diagnosed by following IDF 2006 guidelines among those identified as having metabolic syndrome in this study. But, cases which did not fulfill the obligatory criterion of increased abdominal circumference as per IDF 2006 guidelines could have fulfilled the criteria as per the new Joint Interim Statement 2009. This would have resulted in possibility of increased prevalence of Metabolic Syndrome among COPD patients.

This was a single-centered study conducted on a limited sample size. Our results may not be generalizable to all Nepalese patients. Further studies need to be done with a larger and more representative sample in the future.

## CONCLUSIONS

The study showed that the prevalence of metabolic syndrome was found to be lower than the previous study done in similar settings. So, taking high prevalence in account, screening for metabolic syndrome in COPD patient, and taking appropriate steps can help to reduce deaths in COPD patient due to comorbidities, especially diabetic complications and cardiovascular deaths.
